# Predictors of Health Information–Seeking Behavior: Systematic Literature Review and Network Analysis

**DOI:** 10.2196/21680

**Published:** 2021-07-02

**Authors:** Ardalan Mirzaei, Parisa Aslani, Edward Joseph Luca, Carl Richard Schneider

**Affiliations:** 1 The University of Sydney School of Pharmacy Faculty of Medicine and Health The University of Sydney Australia; 2 The University of Sydney Library The University of Sydney Australia

**Keywords:** information seeking, network analysis, health, review, temporal analysis, mobile phone

## Abstract

**Background:**

People engage in health information–seeking behavior to support health outcomes, and being able to predict such behavior can inform the development of interventions to guide effective health information seeking. Obtaining a comprehensive list of the predictors of health information–seeking behavior through a systematic search of the literature and exploring the interrelationship of these predictors are critical first steps in this process.

**Objective:**

This study aims to identify significant predictors of health information–seeking behavior in the primary literature, develop a common taxonomy for these predictors, and identify the evolution of the concerned research field.

**Methods:**

A systematic search of PsycINFO, Scopus, and PubMed was conducted for all years up to and including December 10, 2019. Quantitative studies identifying significant predictors of health information–seeking behavior were included. Information seeking was broadly defined and not restricted to any source of health information. Data extraction of significant predictors was performed by 2 authors, and network analysis was conducted to observe the relationships between predictors with time.

**Results:**

A total of 9549 articles were retrieved, and after the screening, 344 studies were retained for analysis. A total of 1595 significant predictors were identified. These predictors were categorized into 67 predictor categories, with the most central predictors being age, education, gender, health condition, and financial income. With time, the interrelationship of predictors in the network became denser, with the growth of new predictor grouping reaching saturation (1 new predictor identified) in the past 7 years, despite increasing publication rates.

**Conclusions:**

A common taxonomy was developed to classify 67 significant predictors of health information–seeking behavior. A time-aggregated network method was developed to track the evolution of the research field, showing the maturation of new predictor terms and an increase in primary studies reporting multiple significant predictors of health information–seeking behavior. The literature has evolved with a decreased characterization of novel predictors of health information–seeking behavior. In contrast, we identified a parallel increase in the complexity of predicting health information–seeking behavior, with an increase in the literature describing multiple significant predictors.

## Introduction

### Background

Health information seeking has been defined as “the ways in which individuals obtain information, including information about their health, health promotion activities, risks to one’s health, and illness” [1]. A consumer’s health information–seeking behavior has the potential to influence the process and outcomes related to coping or adjusting to an illness or condition [1].

The conceptualization of health information–seeking behavior has evolved since Lenz first defined it in 1984, which identified 2 dimensions of health information–seeking behavior: extent and method [2]. Lambert and Loiselle’s comprehensive review [1] of the concept of health information–seeking behavior found definitions such as actions or behaviors used to obtain knowledge [3], clarify or confirm knowledge [4], satisfy a query [5], identify information sources [6,7], or demonstrate a coping strategy [8,9]. Other known models from the information science perspective include the Comprehensive Model of Information Seeking, which looks at information carrier characteristics, antecedents, and information-seeking actions [10] and the book by Case in 2002 about the research on information-seeking needs and behaviors [11]. A recent paper by Zimmerman and Shaw describes health information–seeking behavior as an *umbrella term* for many forms of information seeking, such as “direct seeking, information monitoring or browsing, and the passive receipt of information” [12]. Alternatively, people’s passive receipt of health information has been defined separately as *information scanning* [13], where people may not be active in their search but are still receptive enough to receive information. Thus, although the concept of health information–seeking behavior has existed for more than 30 years, there remains a lack of consensus on its definition and model theories.

Despite these inconsistencies, previous models and theories commonly describe health information–seeking behavior as involving the action of seeking out information, regardless of where it comes from, how it is sought, or why it is sought [1,14]. Therefore, predictors of health information–seeking behavior can be described as the variables affecting the actions of seeking out information. These predictors can be contextual, such as the environment of an individual or their social networks, and personal, such as sociodemographic characteristics, health status, or internal beliefs. There may also be predictors of persistence in health information–seeking behavior, such as satisficing. Satisficing relates to decision making “through which an individual decides when an alternative approach or solution is sufficient to meet the individuals’ desired goals rather than pursue the perfect approach” [15]. In the context of information seeking, it is choosing whether it is worth the cost or effort of continuing to search or whether already acquired information suffices.

Factors that influence why people engage in information seeking include the content of the information, which information sources or channels are frequently used, their credibility, and the barriers they may pose to seeking information [1,16]. A meta-analytic review by Chang and Huang quantified 7 predictors of health information–seeking behavior; however, not all of the predictors, such as behaviors (adherence) and beliefs, were included in their review [17]. Predicting peoples’ behavior for health information seeking requires understanding the predictors and their significance and magnitude on information-seeking behavior.

We define *significant predictors* as those shown through empirical research to have a direct effect rather than an association (correlation) to health information seeking. An example of this direct effect is age. Nelissen et al [18] showed that an increase in age led to increased cancer information seeking. An example of an association is the relationship between patient-physician interaction and information-seeking behavior. In this case, it is unclear whether information seeking leads to better patient-physician interaction (an outcome of health information–seeking behavior) or whether better physician-patient interaction leads to increased health information–seeking behavior (a predictor of health information–seeking behavior). Although associations between predictors and health information–seeking behavior may have statistical significance in some empirical studies, knowing the direction of the effect from predictor to variable is more informative. Furthermore, although qualitative research can provide a foundation for identifying predictors of health information–seeking behavior, the ability to quantify the effect size allows for comparison of individual predictors’ relative importance.

A comprehensive list of predictors of health information–seeking behavior provides researchers with a focus on identifying new significant predictors or examining the relationship and effect of new interventions. Predictors of health information–seeking behavior can support researchers, clinicians, private institutions, and public health initiatives in optimizing information-related interactions between themselves and consumers, leading to a more positive health care management experience [1,16].

Consistent classification of terms is the first step in formulating a comprehensive list of health information–seeking behavior predictors. For instance, there is concern that the terms *race* and *ethnicity* have been used synonymously in health research despite being separate constructs [19]. Thus, how predictors of health information–seeking behavior are defined in one study may not necessarily be consistent with another.

Anker et al [16] compiled a comprehensive list of predictors for health information–seeking behavior a decade ago through a systematic search of the literature. They extracted and reported on the methods and measures used in health information–seeking behavior research. However, there are 2 critical shortcomings of this review. First, Anker et al [20] restricted their definition of health information–seeking behavior to an active process, in accordance with Niederdeppe’s definition of health information–seeking behavior. Consequently, predictors for the nonactive acquisition of health information were not identified [20]. Second, their search strategy was restricted to a single database (PsycINFO), with the justification that health information–seeking behavior is a social psychological construct instead of a medical construct. PsycINFO has focused subject areas, and it is possible that other health information–seeking behavior researchers may have published in journals not indexed in PsycINFO.

Importantly, there is a need for an updated review to account for the evolution of information seeking as a result of the rapid emergence and dominance of mobile digital information technology. The use of the internet has been increasing in the past three decades [21]. Advances in technologies such as smartphones have led to increased availability and access to the internet. Since 2011, smartphone ownership by the American population has increased from 35% to 81% in 2019, with 96% of the population owning a cellular device [22]. Similarly, the use of smartphones has led to greater access to the internet, with the exclusive use of smartphones for internet access doubling from a reported 17.5% in 2013 to 37% of the American population in 2019 [21,23]. A smartphone user is estimated to spend a daily average of 2.6 hours on their device [24]. Although the internet has become a common source of health information [25], how the influence of the internet has modified predictors of health information–seeking behavior throughout time is yet to be well characterized. Previous studies have compared sources of information used by people as part of health information–seeking behavior; however, most studies have only compared the findings from the early 2000s with those from the early 2000s [26-28]. Such comparison studies report that internet use was not a predictor of information-seeking behavior, yet Huerta et al [26] reported an increase in internet use, especially in older age groups. In contrast, Li et al [28] performed a hierarchical regression analysis comparing 2002 and 2012 cohorts from the Pew Database to examine changes in health information–seeking behavior on the web. They identified internet access as a predictor for health information decline with time. The authors hypothesized that this could be partly because of the increase in misinformation and rise in smartphone use, resulting in increased accessibility of the internet as a source of health information in the United States. The extent to which these changes have affected predictors of health information–seeking behavior as a whole and across information sources and settings has yet to be reviewed.

### Objectives

This review aims to identify predictors of health information–seeking behavior, as reported in the primary literature, and explore the relationships between predictors with time. The specific objectives are as follows:

- identify significant predictors of health information–seeking behavior in the primary literature;
- develop a common taxonomy for predictors of health information–seeking behavior;
- identify the evolution of the health information–seeking behavior research field using quantitative studies.

## Methods

### Selection Criteria

The following section outlines the inclusion and exclusion criteria for this review.

#### Types of Participants

The papers included defined participants as health consumers or caregivers. The intent of the search for information was important: a health consumer searches for information for their own self or treatment, as opposed to a health professional who may search for information to provide therapy. Caregivers were also included, as they were in a nontherapeutic relationship with the health consumer.

Papers in which the participants were health students (university or college) or simulation studies in which the participants sought information prospectively in hypothetical scenarios were excluded. Students studying a health-related degree were removed if in their health-related disciplines, they were searching for information for their future role as health professionals.

#### Types of Studies

Quantitative studies were eligible for inclusion in this study. Relevant study designs included experimental and epidemiological studies, including randomized controlled trials, nonrandomized controlled trials, quasi-experimental studies, before-and-after studies, prospective and retrospective cohort studies, case-control studies, and analytical cross-sectional studies.

An article was included if it reported the significance level of a predictor. That is, the study showed that a certain *predictor* was significant in causing *information seeking* rather than associations. Significance was determined by either *P* values <.05 or in the case of odds ratio if the CI did not cross 1. Articles were also included if further work demonstrated a causation or effect size, which was often shown through logistic or linear regression, confirmatory factor analysis, or structural equation modeling.

Studies were excluded if they did not have a quantitative focus. These included qualitative studies such as focus groups, semistructured interviews, mixed methods studies (with no quantitative component), and content analysis of websites or interviews.

### Search Procedures

#### Search Limits

Papers published in English up to December 10, 2019, since database inception were considered for inclusion. No data range was applied. Participants’ information seeking was not restricted to any source, and all sources (eg, web, health care practitioner) were included.

#### Databases

The databases searched were PsycINFO, Scopus, and PubMed. Scopus is considered the largest abstract and citation database of peer-reviewed literature and incorporates the results from Embase and MEDLINE [29].

#### Search Terms or Phrases

The keywords used were the following:

*Health* OR *Drug* OR *Medicine*; AND *Information Seeking* OR *Information Behavior* OR *Information Search* or *Satisficing* (Tables S1-S3 in Multimedia Appendix 1 for full syntax).

#### Screening

Two authors (AM and EJL) independently screened a random split of articles for inclusion by title and abstract using the selection criteria. Pilot tests were conducted to calibrate the screening process before the records were split.

### Data Extraction

Each included paper was counted as a data source for the extraction; 2 authors (AM and EJL) independently extracted the following variables: year of publication, country of the study, participant recruitment, disease states, theories used, and significant predictors. Significant predictors were those variables for which direct effects were reported (not correlations) on health information–seeking behavior and provided significance with either *P* values <.05 or CIs (in the case of odds ratio) that did not cross 1. Significant relative predictors of health information–seeking behavior were also extracted from studies reporting comparative health information–seeking behavior between 2 or more groups. Any uncertainty associated with extraction was mediated by a third author (CRS), who performed an audit of approximately 10% (34/344) of the screened articles and extracted variables.

#### Analysis

##### Content Analysis

The first author (AM) analyzed the variables and categorized the significant predictors into emerging categories. Individual predictors identified in the individual papers were extracted, and an iterative process of clustering was undertaken by 2 authors (AM and CRS); the 2 authors reached a consensus for terms and categories. The categories consisted of identifying similarity between predictor terms: where predictors were the same, they were categorized together; where predictors were similar with a common definition, they were categorized together; and finally, where there was no common definition, but predictors were described similarly in text, they were categorized together [30].

##### Predictor Frequency

As part of the content analysis, a *word frequency* analysis was performed. In this case, the *words* chosen were identified-predictor terms. Examining the predictor term frequency assists in analyzing the strength and importance of a predictor with regard to other terms [31-34]. Each predictor extracted into a category was counted as 1 for that article. Multiple predictors, if categorized, were categorized as 1. For example, if the article reported “Age 25-30” and “Age 65-70” as significant factors, then they would be categorized as *age*; however, they would contribute only 1 to the *age* category for that article instead of 2. The total predictors were then reported, and the predictor frequency was used to develop the network structure for network analysis.

#### Network Analysis

Network analysis has been used in previous systematic reviews to identify relationships among authors of the included papers [35] or in a health context to compare drug treatments [36]. Traditionally, quantitative data from a systematic review are pooled via meta-analysis, which requires homogeneous data. Network analysis allows the examination of relationships among heterogeneous entities [37]. A network analysis was conducted to observe the relationships between the predictor terms. This method can help identify nodes (or predictor terms) connected to other nodes and show the relationships between terms in the literature [38,39].

The weights of the nodes were based on the frequency of the predictor, whereas their size was based on the number of articles mentioning the predictor term. An analysis of changes throughout time was undertaken to compare the networks of articles before and after 2008 (articles dated up to December 31, 2008)—while Pew Research started reporting smartphone ownership in 2011 [22], the iPhone was the beginning of a new phone era [40]. The year 2008 was chosen to distinguish between the availability of smartphones following the introduction of the iPhone in 2007, allowing for the uptake of the device to have begun [41]. Accordingly, in 2008, global mobile broadband subscriptions overtook fixed broadband subscriptions [42]. Time-based comparisons of temporal and atemporal network features were observed using time-varying networks. Such an approach has been used in ecology, transport, and social media [43-45].

The co-occurrence of individual predictors within an article was calculated based on the predictor frequency. Each individual predictor term was connected bidirectionally to another predictor from the same article. Each connection adds a weight of 1 to the edge. Edges were formed where a pair of predictor terms was mentioned together in an article. The visualization was created using R software (R Foundation for Statistical Computing), with the code available on GitHub [46]. The co-occurrence of predictors and visualization of the network was created using the igraph package, a software package used for network visualizations between different objects on a network map [47].

The number of nodes and edges, along with modularity, were captured to compare the different networks. Modularity measures the clustered communities of nodes, which is how the nodes cluster together, forming a community group of nodes distant to another community group of nodes. The full setup and parameters are available in GitHub [46]. See Multimedia Appendix 1 for further methods [38,39,48,49]

## Results

### Search Results

**Figure 1:**
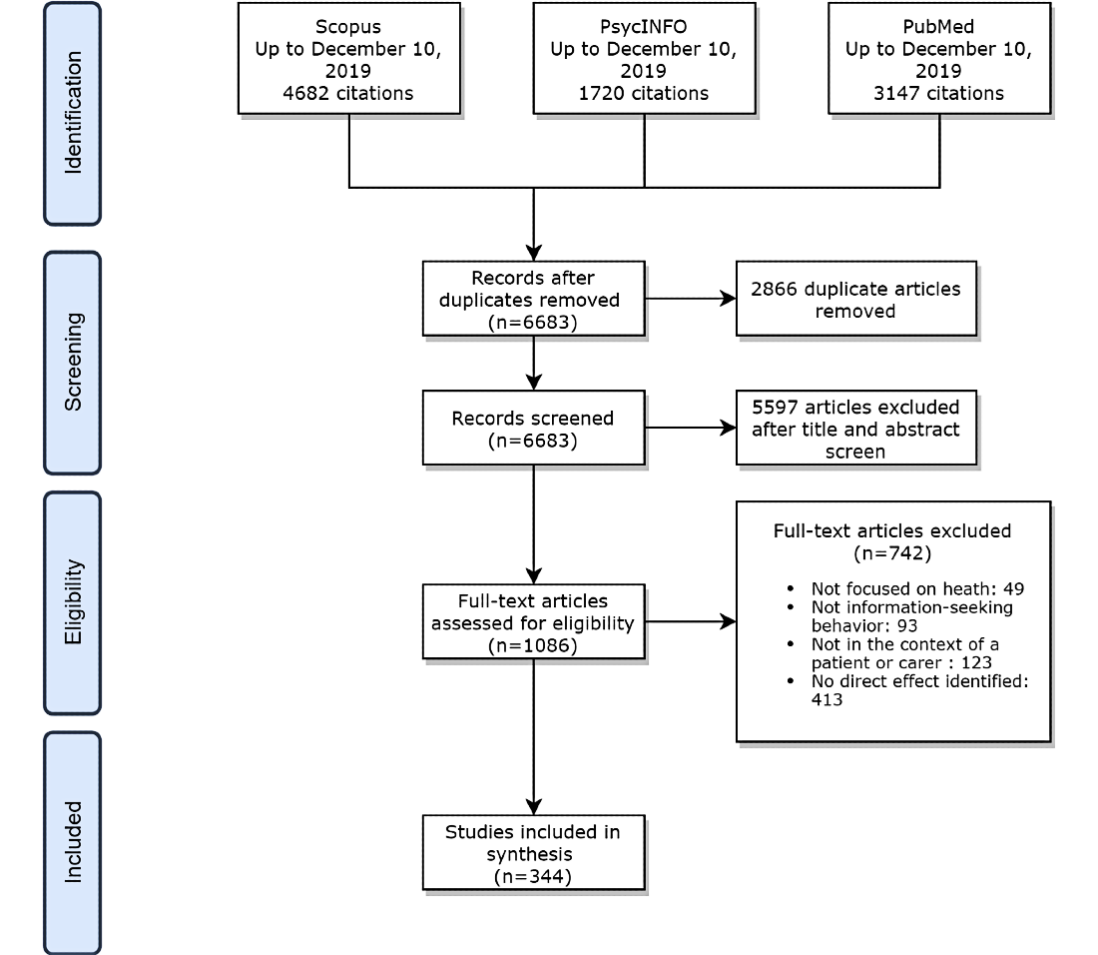
Flowchart for article inclusion. Reasons for exclusion do not sum to the number of excluded articles because some articles overlapped in their reasons for exclusion.

**Figure 2:**
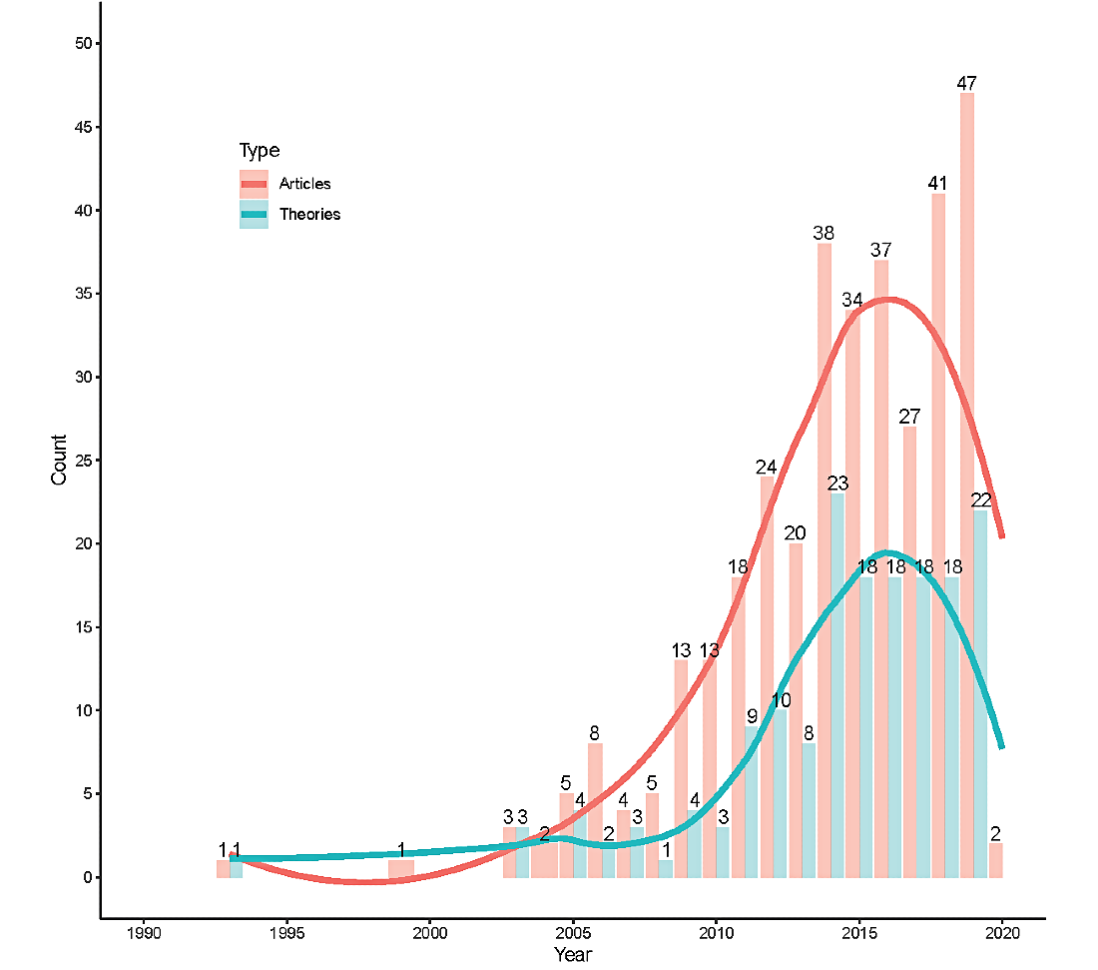
Total articles published each year and the number of articles that used a theoretically grounded approach.

The literature search process is illustrated in Figure 1. From the 2 databases, a total of 9549 articles were retrieved, of which 2866 were duplicates. Following deduplication, title and abstract screening was performed, followed by full-text screening. A total of 344 papers were included in the final analysis. The results of the categorized predictors are reported in Table S4 in Multimedia Appendix 1. The included articles contained papers published between 1993 and 2019, with a peak publication year in 2019 (Figure 2).

Most of the studies were conducted in the United States (n=202); 26 articles reported studies from China, 12 from Australia and South Korea, and 9 from the United Kingdom and Germany.

In 203 articles, participants were recruited specifically for the study. However, another source of participants was using existing databases of respondents such as the Health Information Trends Survey (HINTS; n=65 papers), The Pew Research Center’s Internet & American Life Project (n=9), and the Pennsylvania Cancer Registry (n=8).

Participants were predominantly seeking information for chronic diseases, with cancer being the most studied condition (n=76).

### Content Analysis

Fewer than half of the papers (n=167) were underpinned by a theory or model. As shown in Figure 2, there was an increase in the number of papers with a theoretical underpinning until 2014, at which point a plateau developed from 2015 to 2018. In 2019, there was a second peak of articles with theoretical underpinnings. However, less than half of the papers published in 2019 were supported by a theory or model (Figure 2).

A total of 1595 nonunique, significant predictors were identified. Table S4 in Multimedia Appendix 1 lists the predictor categories. Predictors were classified into 68 categories (1 category was labeled *unclassifiable/other*; unclassifiable predictors were not carried forward for network analysis.). The categories were further grouped into sociodemographic, health, information source, information content, and affective predictor groups. Sociodemographic variables of education (n=160), age (n=156), and gender (n=120) were the most commonly reported significant predictor categories, followed by the health-related predictor categories of health condition (n=87). A noted increase in the number of predictors reported in the literature began in 2005 and peaked in 2019.

### Network Analysis

The complete network with all terms and years combined resulted in 67 nodes (*other* node not included) and 4128 edge connections. The modularity of the groups revealed 3 clusters. The largest group of variables was predominately composed of psychosocial predictors (n=41). The second-largest group was sociodemographic predictors (n=22), followed by a third group that did not have any strong focus on any particular grouping (n=4). However, most sociodemographic variables (age, education, gender) had the greatest eigenvector centrality before other variables (health condition and financial income), thus appearing in the center of the network (Table S5 in Multimedia Appendix 1).

**Figure 3:**
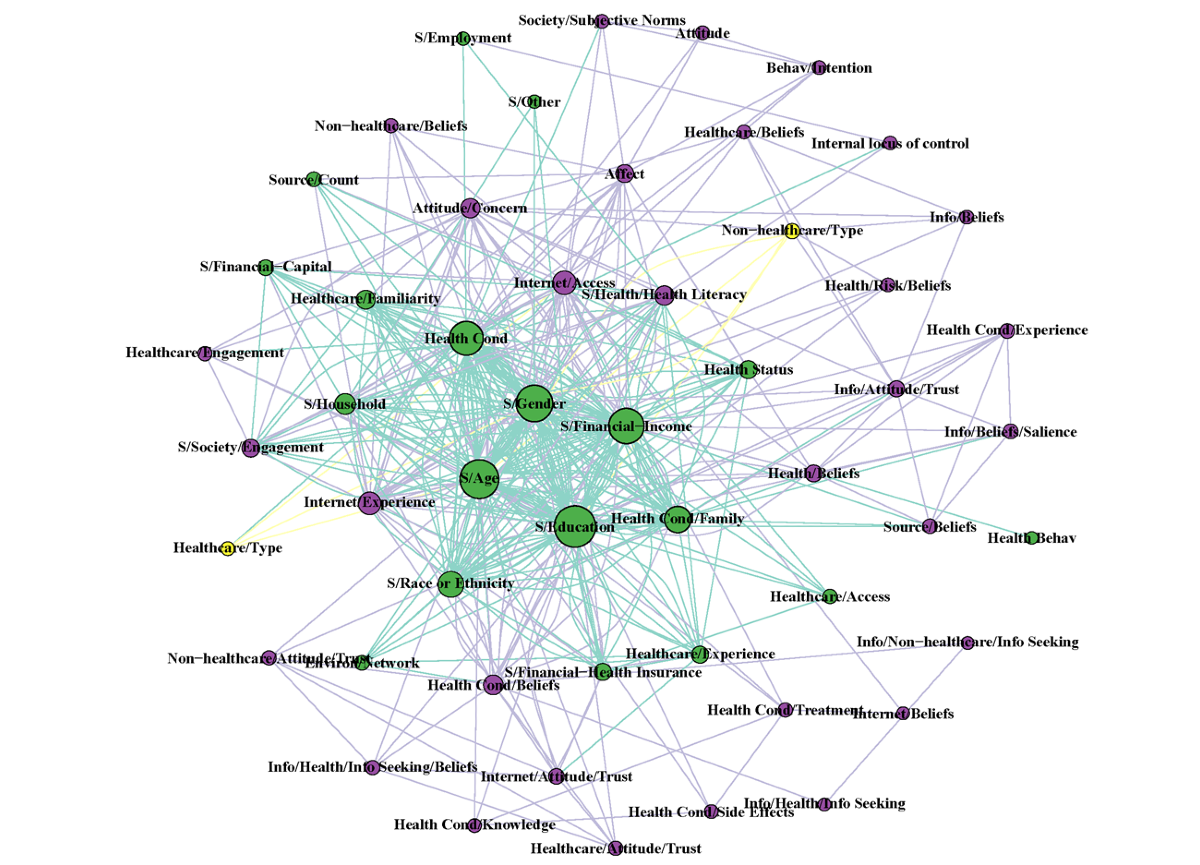
Fruchterman-Reingold layout algorithm of the network analysis comparing the pre- and including 2008 network models with color coding according to the modularity group membership of the complete model. The repository is available on GitHub [46].

**Figure 4:**
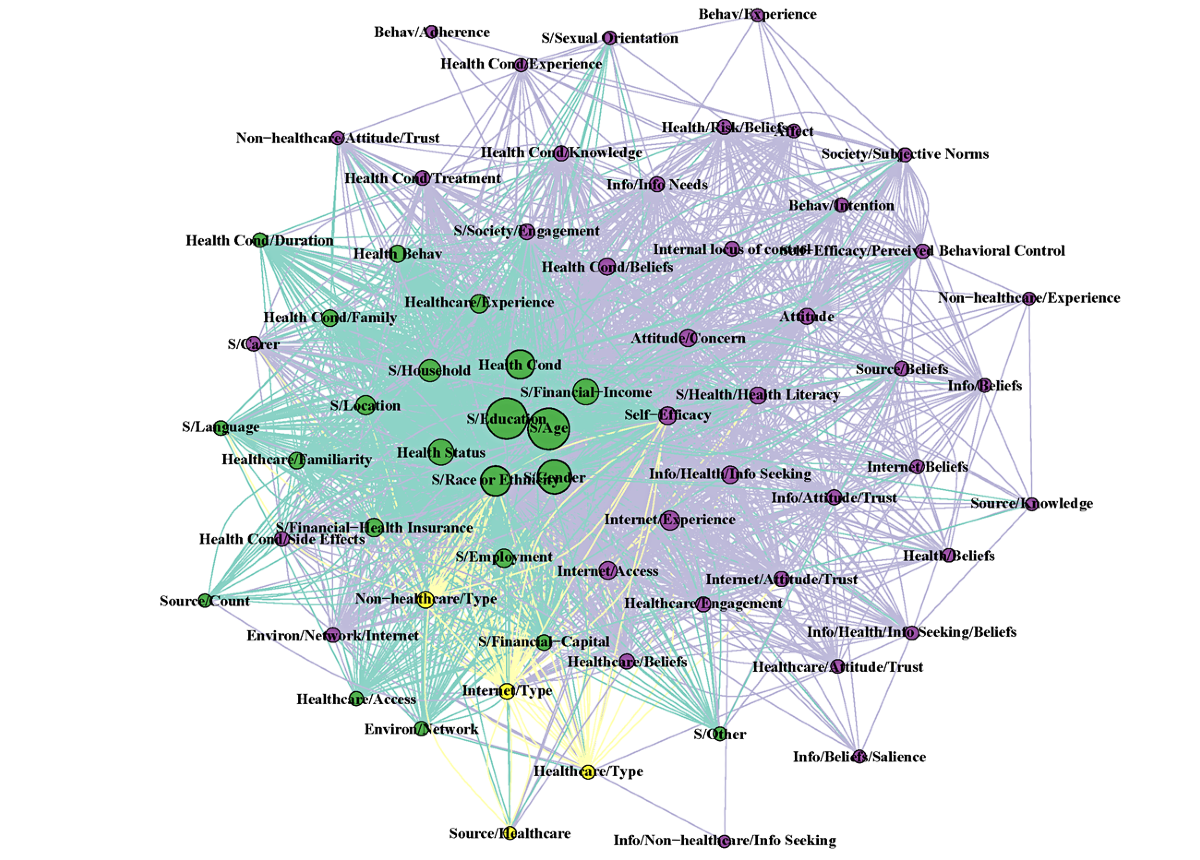
Fruchterman-Reingold layout algorithm of the network analysis comparing the post-2008 network model with color coding according to the modularity group membership of the complete model. The repository is available on GitHub [46].

**Figure 5:**
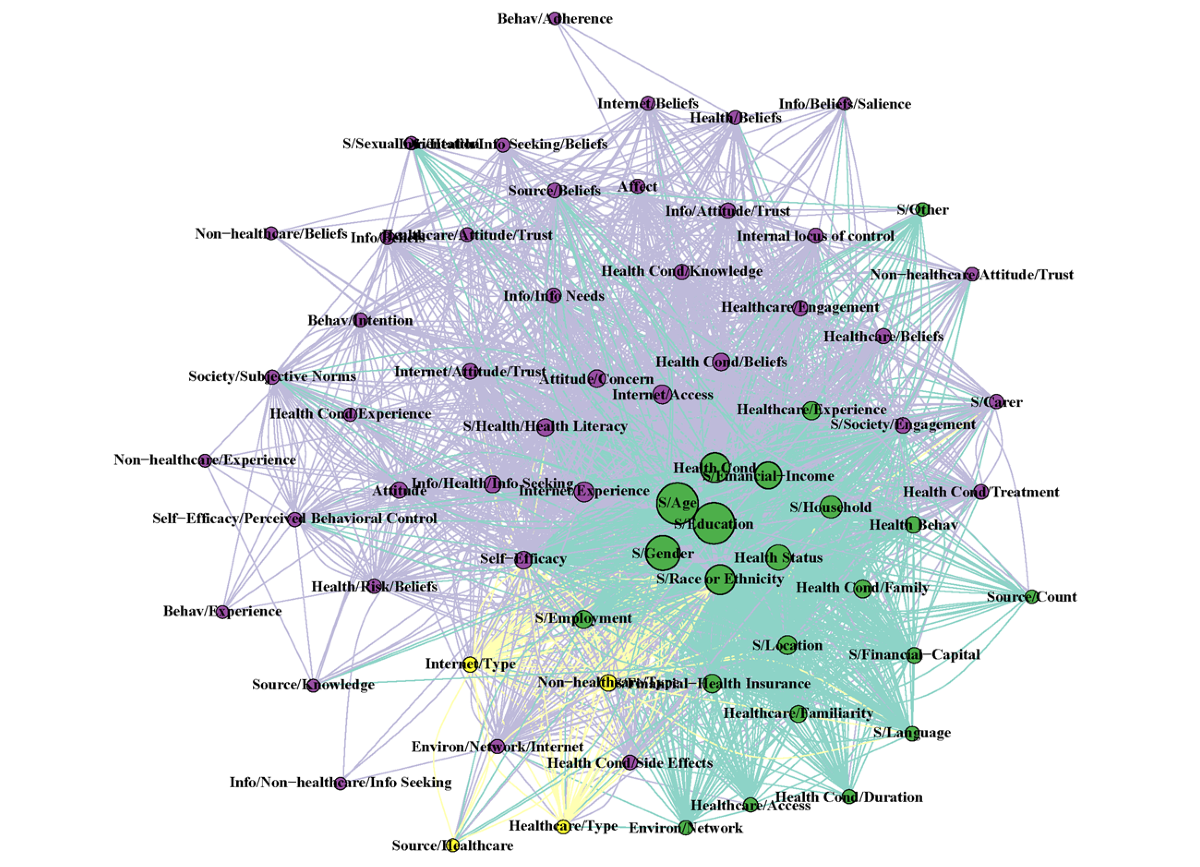
Fruchterman-Reingold layout algorithm of the network analysis comparing the complete model with all years until 2019 with color coding according to the modularity group membership of the complete model. The repository is available on GitHub [46].

The network statistics reported in Table S5 of Multimedia Appendix 1 as well as in Figures 3 and 4 show a difference in structural characteristics before and after 2008. After 2008, only 15 new nodes were identified, with no new nodes identified after 2014 (Table S4 in Multimedia Appendix 1). There was a 7.8 times greater number of edges in the post-2008 network than in the pre-2008 network. The combination of an increased number of edges and a limited increase in nodes resulted in a more connected network after 2008, with the average number of adjacent edges to each node (mean degree of the nodes) increased by 6.1 times compared with before 2008. Age, education, gender, and health condition were the nodes with the greatest degree of centrality, indicating the greatest influence on adjacent nodes (Figure 5). Modularity was greatest before 2008; it decreased to 2 in the post-2008 period, indicating tighter communities of nodes clustering together, with all years compared being 3. This tighter clustering is because of the greater co-occurrence of the predictor terms being researched. That is, individual articles reported more significant predictors than previous articles. A sensitivity analysis was conducted to compare networks before and after 2014 (the most recent new node), which confirmed the dynamic shifts in network statistics after 2008 (Table S5 in Multimedia Appendix 1).

## Discussion

### Principal Findings

This paper reports a systematic literature search to identify and characterize predictors of health information–seeking behavior. The 344 included papers report 1595 significant predictor terms of health information–seeking behavior that can be classified into 68 categories. A comprehensive list of health information–seeking behavior predictor terms was developed. A novel temporal network analysis through the comparison of 2 sequential time-aggregated networks was conducted to characterize the relationships between health information–seeking behavior predictors and identify changes throughout time. This approach has never been previously used to characterize such relationships. Key findings were an increase in papers reporting on multiple significant predictors of health information–seeking behavior within a paper and a reduced rate in the identification of new predictors. The use of network analysis to map the relationships within a research field throughout time demonstrates the evolving nature of research and provides insight into how the understanding of predictors of health information–seeking behavior has developed. Van de Wijngaert et al [50] conducted a network approach to examine the current state in a research field pertaining to the adoption of eGovernment services. However, they used structural equation modeling and cross-sectional analysis, as opposed to our network comparison. The advantage of network comparison throughout time is that it allows for characterizing the evolution of research fields. Future work would be to explore the use of temporal dynamics of networks, ideally through the analysis of longitudinal data sets [43-45].

A recent meta-analysis conducted by Chang and Huang [17] on the antecedents predicting health information seeking aggregated the antecedents into 7 categories; through their review, they were able to quantify the effect sizes of the 7 categories. However, in their methodological design, not all the predictors of health information–seeking behavior were captured. Although a valuable review, we note the differences in design and the papers that could be retrieved. Since the initial review by Anker et al [16] in 2010, an increased number of papers exploring predictors of health information–seeking behavior has allowed for greater granularity in the identification of health information–seeking behavior predictors. Specifically, we have been able to develop a sociodemographic group of predictors from 5 predictors (age, gender, education, race, and health literacy) to 16 predictors (caregiver, employment, household, language, sexual orientation, finances, societal engagement, and location of residence). A potential benefit of such granularity is the improved targeting of interventions to optimize health information–seeking behavior.

Anker et al [16] reported that medication adherence is an outcome of engaging in health information seeking. However, according to our review, adherence was identified as a predictor. This suggests that health information–seeking behavior is affected by a feedback loop, where outcomes from health information–seeking behavior can be a predictor for further health information–seeking behavior. This relationship should be examined in longitudinal studies. Few longitudinal studies have examined health information–seeking behavior, but our findings suggest that this could lead to unidentified health information–seeking behavior predictors. A longitudinal study described the reciprocal relationship between health anxiety and health information–seeking behavior on the web and how *cyberchondriac* people have health anxiety exacerbated [51]. Another study looked at clinician information engagement and information seeking [52], whereas others have shown that information needs and preferences change with time [53,54]. These initial findings demonstrate the utility of further longitudinal studies to measure additional predictors and outcomes of health information–seeking behavior.

The rate of article publication on health information–seeking behavior increased after 2005, with a doubling of articles published in the past 10 years compared with the prior 30 years. This finding mirrors the overall increase in the academic publishing rate [55]. Another explanation might be the establishment of data gathering institutions, such as the HINTS from the National Cancer Institute, which was established in 2003. Such cohort studies provide researchers with important opportunities to examine health information–seeking behaviors across large nationally representative sampling frames. HINTS is the most used data set across papers; therefore, it is the main source of identified health information–seeking behavior predictors. HINTS comprises 12 cross-sectional surveys that have been conducted in the past 15 years [56-65]. The data set has the advantage of being a representative sample frame of the United States.

The number of articles using theory to underpin their research has also increased with time. The use of theory has become a consistent theme in describing significant predictors. Interestingly, in the past 7 years, there has been a plateau in the frequency of publications reporting health information–seeking behavior predictors. A possible reason for this is maturation in the literature, with apparent saturation of identified predictors. Li et al [66] also identified an increase in publications until 2014. Our findings have extended this trend to demonstrate a plateau in publication rates since 2015.

Participants’ interactions with social media, including social networking sites and health blogs, which were categorized as environment/network/internet, were identified as new predictors since 2008. Hamid et al [67] reviewed the role of social media in information-seeking behavior among international students, highlighting that specific information needs were satisfied by using social media. Although social media can be a medium for public health intervention [68], it can also present a challenge as a source of misinformation [69,70]. Competing misinformation has implications for providers of information using web media to target their audience. Providers or information creators could address the rise of misinformation by ensuring that the content delivered through social media is verified for quality and that continued monitoring is implemented.

The terminology used to describe predictors varied significantly between papers and, at times, lacked precision. Articles may have mentioned race as a predictor, but on closer inspection of the survey, items used for race, ethnicity, and culture overlap. A potential contributor to the lack of clarity in terminology is the low number of studies that used a theoretically grounded approach. Ambiguous terminology poses a challenge when comparing findings between papers on health information–seeking behavior. In response to this issue, this review developed a common classification structure for predictor terms. This structure has the potential to be developed into a future consensus taxonomy for predictors of health information–seeking behavior using domain ontologies [71].

Conducting a network analysis for predictors of health information–seeking behavior is a novel approach for analyzing the health information–seeking behavior research field. The overall network analysis shows the interrelationship of the predictor variables; however, the interaction between these variables in predicting health information–seeking behavior is still unclear. A concern is the issue of terms being correlated with each other, resulting in collinearity. The collinearity of predictor terms may affect how an individual predictor term affects the health information–seeking behavior. The temporal network analysis finding of a 6.1 greater mean degree demonstrates an increase in publications reporting multiple significant predictors. Increased reporting was also supported by an increase in co-reporting, represented by reducing network modularity with time. The high centrality of age, education, gender, and health condition indicates that these were the most commonly reported predictors when multiple predictors were reported in the included studies. Such studies have a greater ability to identify the predictors of collinearity. The network analysis approach allowed us to examine how our understanding of predictors of health information–seeking behavior and their interrelationships changed with time. Such changes have occurred in the presence of a shift toward mobile technology becoming commonplace.

### Strengths and Limitations

This review has several strengths. Multiple authors followed a rigorous methodology to extract data and reach an agreement on definitions. However, because of the sheer number of articles returned in the initial database searches, there is a potential risk that articles meeting the inclusion criteria could have been potentially omitted. Nevertheless, the likelihood of omitted articles affecting the findings of this review was low because of the number of included articles. The developed taxonomy of predictors was directly informed by the included articles via a theoretical agnostic approach and consensus between 2 authors (AM and CRS). The content validity of the developed taxonomy would benefit from validation via conceptual synthesis and a consensus approach from experts in the field of health information–seeking behavior. The reliability of data extraction could be considered a limitation of our review, which we tried to mitigate through a 10% audit by a third author (CRS). Using a quantitative measure of intercoder reliability would increase confidence in reliability.

Limitations to the search strategy are, first, the inclusion of only articles published in English. This is a potential issue in this field, as geographic and cultural differences have been identified. The United States is the most represented country. However, a bibliometric analysis of the internet health information–seeking behavior literature has been previously performed by Li et al [66]. The authors similarly identified a majority of articles from the United States. A skew toward a single country may introduce geographic bias in the literature and subsequent identification of significant health information–seeking behavior predictors. There is evidence that context can directly influence individuals’ health information–seeking behavior, such as being in a low-resource setting [72]. Therefore, it is important to be mindful of the number of studies from high-resource settings when considering the implications of our findings in low-resource settings. For instance, the presence of the predictor variable *health care source accessibility* may be more pertinent for countries without universal health care coverage, such as the United States, where access to physicians is variable [73,74] (Multimedia Appendix 1). A second limitation is the restriction of the definition of health information–seeking behavior as an active behavior. This limited the ability of the review findings to represent the predictors of passive health information–seeking behavior or *scanning*. Third, the review findings do not represent predictors of health information–seeking behavior for university students because of the exclusion of this subpopulation.

Finally, systematic reviews typically include an assessment of the risk of bias. The heterogeneity of the studies and the observational nature of most study designs meant that an assessment of bias was not appropriate. This led to this systematic review not adhering to the PRISMA (Preferred Reporting Items for Systematic Reviews and Meta-analyses) guidelines and protocols in their entirety. However, the systematic approach adds to the strengths of this study.

### Conclusions

A systematic literature search identified 344 papers reporting the predictors of health information–seeking behavior. A common taxonomy was developed to classify the predictors of health information–seeking behavior into 67 categories. Only 24% (16/67) of the predictor groupings have emerged since the invention of smartphones. Novel network analysis identified that the growth of new predictor groupings had approached saturation with only a single new predictor identified in the past 7 years, despite increasing publication rates. Publication network analysis is a promising methodology for measuring trends across scientific fields.

## Appendix

Multimedia Appendix 1 Search terms, list of predictors and their definitions, network statistics, and network analysis methods.

## Abbreviations

HINTS: Health Information Trends Survey
PRISMA: Preferred Reporting Items for Systematic Reviews and Meta-analyses
