# Correction: Predictors of Health Information–Seeking Behavior: Systematic Literature Review and Network Analysis

**DOI:** 10.2196/39705

**Published:** 2022-06-03

**Authors:** Ardalan Mirzaei, Parisa Aslani, Edward Joseph Luca, Carl Richard Schneider

**Affiliations:** 1 School of Pharmacy Faculty of Medicine and Health University of Sydney Sydney Australia; 2 The University of Sydney Library Sydney Australia

In “Predictors of Health Information–Seeking Behavior: Systematic Literature Review and Network Analysis” (J Med Internet Res 2021;23(7):e21680) the authors noted three errors.

1. In the originally published article, Reference 11 was incorrectly published as follows:

Cole C. Looking for information: a survey of research on information seeking, needs, and behavior (4th edition). Donald O. Case and Lisa M. Given. Bingley, UK: Emerald Group Publishing, 2016. 528 pp. $82.95 (hardcover). (ISBN: 9781785609688). J Asso Inf Sci Technol 2016 Dec 21;68(9):2284-2286. [doi: 10.1002/asi.23778]

The correct reference is a book and has been updated as follows:

Case DO, Given LM. Looking for information: a survey of research on information seeking, needs, and behavior (4th edition). Bingley, UK: Emerald Group Publishing; 2016.

2. The in-text citation for reference 11 incorrectly mentioned the year as 2002 in the following sentence:

Other known models from the information science perspective include the Comprehensive Model of Information Seeking, which looks at information carrier characteristics, antecedents, and information-seeking actions [10] and the book by Case in 2002 about the research on information-seeking needs and behaviors [11].

The correct year for the current edition of the book is 2016 and the sentence has been updated as follows:

Other known models from the information science perspective include the Comprehensive Model of Information Seeking, which looks at information carrier characteristics, antecedents, and information-seeking actions [10] and the book by Case in 2016 about the research on information-seeking needs and behaviors [11].

3. In affiliations 1 and 2 of the originally published article, the city was incorrectly mentioned as 'The University of Sydney'. This has been corrected to 'Sydney,' and the correct list of affiliations is as follows:

Ardalan Mirzaei^1*^, BPharm, MPhil, GCertEdStud (Higher Ed); Parisa Aslani^1*^, BPharm, MSc, PhD, GCertEdStud (Higher Ed); Edward Joseph Luca^2^, BA, MBA; Carl Richard Schneider^1*^, BN, BPharm, PhD, PGCert (Higher Ed)

^1^School of Pharmacy, Faculty of Medicine and Health, University of Sydney, Sydney, Australia

^2^The University of Sydney Library, The University of Sydney, Sydney, Australia

*these authors contributed equally

The correction will appear in the online version of the paper on the JMIR Publications website on June 3, 2022, together with the publication of this correction notice. Because this was made after submission to PubMed, PubMed Central, and other full-text repositories, the corrected article has also been resubmitted to those repositories.

