# Supplementary material for: Predictors of Health Information–Seeking Behavior: Systematic Literature Review and Network Analysis
Source: J Med Internet Res. 2021 Jul 2;23(7):e21680. doi: 10.2196/21680 (PMC8285748; doi:10.2196/21680)
Supplement: Multimedia Appendix 1 [file jmir_v23i7e21680_app1.docx]

## Supplementary Material

### Table 1, 2 and 3: Search Terms

Table S1: Scopus search method and terms

| 1 | ( ( TITLE-ABS-KEY ( ( "information seeking " OR "information behavio?r" OR satisfic* OR "information search*" ) ) ) AND ( TITLE-ABS-KEY ( ( "health" OR "medic*" OR "drug*" ) ) ) ) AND NOT ( TITLE-ABS-KEY ( ( qualitativ* ) AND ( "focus group*" ) ) ) AND ( LIMIT-TO ( DOCTYPE , "ar" ) OR LIMIT-TO ( DOCTYPE , "re" ) ) AND ( LIMIT-TO ( LANGUAGE , "English" ) ) |
| --- | --- |

Table S2: PsycINFO search method and terms

| 1 | exp HEALTH/ |
| --- | --- |
| 2 | health.mp. |
| 3 | exp DRUGS/ |
| 4 | medic*.mp. |
| 5 | drug*.mp. |
| 6 | exp Information Seeking/ |
| 7 | information seeking.mp. |
| 8 | information behavio?r.mp. |
| 9 | satisfic*.mp. |
| 10 | Information search*.mp. |
| 11 | 1 or 2 or 3 or 4 or 5 |
| 12 | 6 or 7 or 8 or 9 or 10 |
| 13 | focus group*.mp. |
| 14 | qualitativ*.mp. |
| 15 | exp QUALITATIVE RESEARCH/ |
| 16 | 13 or 14 or 15 |
| 17 | 11 and 12 |
| 18 | 17 not 16 |
| 19 | limit 18 to english language |

Table S3: PubNMed search method and terms

| 1 | (("health"[MeSH Terms] OR "health"[All Fields] OR "health s"[All Fields] OR "healthful"[All Fields] OR "healthfulness"[All Fields] OR "healths"[All Fields] OR "drug*"[All Fields] OR "medic*"[All Fields]) AND "english"[Language]) AND "english"[Language]) AND (("information seeking"[All Fields] OR "information behavio*"[All Fields] OR "satisfic*"[All Fields] OR "information search"[All Fields]) AND "english"[Language]) AND "english"[Language]))) NOT (((("wood des focus"[Journal] OR "focus"[Journal] OR "focus ohio dent"[Journal] OR "focus madison"[Journal] OR "focus am psychiatr publ"[Journal] OR "focus"[All Fields]) AND "group*"[All Fields]) OR "qualitativ*"[All Fields] OR "qualitative research"[All Fields]) AND "english"[Language])) |
| --- | --- |

### Table 4: Predictors and definitions

Table S4: Definition and first year of mention of identified significant predictors

| Name | Definition | Year |
| --- | --- | --- |
| Affect | Affect is the feelings that an individual will possess | 2003 |
| Affect/Attitude | Degree to which a person has a favorable or unfavorable evaluation or appraisal of the proposition in question | 2005 |
| Affect/Attitude/Concern | Degree to which a person has an unfavorable evaluation or appraisal of the proposition in question | 2003 |
| Affect/Internal locus of control | Health locus of control refers to persons’ beliefs about whether they have internal control over their own health or their health is determined by external factors. | 2006 |
| Affect/Self-Efficacy | Beliefs in one's capabilities to organize and execute the courses of action required to manage prospective situations | 2009 |
| Affect/Self-Efficacy/Perceived Behavioral Control | Perceived behavioral control is often assessed by the ease or difficulty of the behavior | 2011 |
| Behavior/Adherence | Persistence in a practice or tenet; steady observance or maintenance to a therapeutic regimen | 2011 |
| Behavior/Experience | Prior performance of the behavior | 2014 |
| Behavior/Intention | Intentions are assumed to capture the motivational factors that influence a behavior; they are indications of how hard people are willing to try, of how much of an effort they are planning to exert, in order to perform the behavior. | 2005 |
| Environment/Network | Community network size and grouping | 2005 |
| Environment/Network/Internet | Online communities and groups | 2011 |
| Health/Beliefs | Health Belief is the cognitive act or state in which a belief about health in general is taken to be true | 1993 |
| Health/Health Behavior | Performing an action related to a health proposition | 2004 |
| Health/Health Condition | The presence of a medical or health condition | 2003 |
| Health/Health Condition/Beliefs | The cognitive act or state in which a belief about a health condition is taken to be true | 2005 |
| Health/Health Condition/Duration | Duration for a health condition or time since diagnosis. | 2009 |
| Health/Health Condition/Experience | Prior experience to the health condition | 1993 |
| Health/Health Condition/Family | Indirect exposure to a health condition through a family member | 1993 |
| Health/Health Condition/Knowledge | The level of knowledge an individual possesses of a health condition. | 2005 |
| Health/Health Condition/Side Effects | The presence of side effects | 2005 |
| Health/Health Condition/Treatment | Therapeutic care, product or regimen for a health condition | 2005 |
| Health/Health Status | A self-rated measure of an individual's perception of their health | 2003 |
| Health/Risk/Beliefs | Health risk belief is the cognitive act or state in which a belief about health risks are taken to be true | 1999 |
| Information/Attitude/Trust | Trust incorporates reliability, credibility, perceived quality, and confidence in the information | 1993 |
| Information/Beliefs | Information belief is the cognitive act or state in which a belief about information is taken to be true | 2008 |
| Information/Beliefs/Salience | Salience to an individual means the perceived applicability of information to a problem that he or she faces. | 1993 |
| Information/Health/Information Seeking | Purposive acquisition of health information | 2007 |
| Information/Health/Information Seeking/Beliefs | Information seeking belief is the cognitive act or state in which a belief about health information seeking is taken to be true | 2007 |
| Information/Information Needs | Information needs of an individual | 2009 |
| Information/Non-healthcare/Information Seeking | Purposive acquisition of general information not related to health | 2007 |
| Non-healthcare/Beliefs | Non-Health belief is introduced as the cognitive act or state in which a non-health related proposition is taken to be true | 2003 |
| Society/Subjective Norms | Refers to the perceived social pressure to perform or not to perform the behavior | 2005 |
| Sociodemographic/Age | The age of an individual | 1999 |
| Sociodemographic/Carer | Carer status | 2009 |
| Sociodemographic/Education | An individual’s educational attainment | 1993 |
| Sociodemographic/Employment | Occupation and employment of an individual | 2006 |
| Sociodemographic/Financial-Capital | The financial capital of an individual | 2008 |
| Sociodemographic/Financial-Health Insurance | Insurance coverage for health | 2006 |
| Sociodemographic/Financial-Income | An individual’s personal or household income | 1993 |
| Sociodemographic/Gender | Gender | 1999 |
| Sociodemographic/Health/Health Literacy | Health literacy is the degree to which individuals have the capacity to obtain, process, and understand basic health information and services | 2005 |
| Sociodemographic/Household | Type and size of an individual’s family including marital status | 2006 |
| Sociodemographic/Language | Languages spoken | 2009 |
| Sociodemographic/Location | Where an individual lives | 2009 |
| Sociodemographic/Other | Sociodemographic variable not classified elsewhere | 2006 |
| Sociodemographic/Race or Ethnicity | Race, culture or ethnicity of an individual | 2005 |
| Sociodemographic/Sexual Orientation | The sexual orientation of the individual | 2009 |
| Sociodemographic/Society/Engagement | Integration and engagement with social support or society | 2003 |
| Source/Beliefs | The cognitive act or state in which a belief about an unspecified type of information source is taken to be true | 1993 |
| Source/Count | The number of sources used for information | 2004 |
| Source/Healthcare | A healthcare source of information | 2012 |
| Source/Healthcare/Access | Accessibility of the healthcare source | 2006 |
| Source/Healthcare/Attitude/Trust | Trust incorporates reliability, credibility, perceived quality, and confidence towards a healthcare information source. | 2007 |
| Source/Healthcare/Beliefs | Healthcare source is the cognitive act or state in which a belief about the healthcare provider is taken to be true | 2008 |
| Source/Healthcare/Engagement | Engagement with healthcare information sources | 2003 |
| Source/Healthcare/Experience | Previous exposure to the healthcare source of information | 2005 |
| Source/Healthcare/Familiarity | Usual or regular source of healthcare | 2003 |
| Source/Healthcare/Type | Type of healthcare information source | 2005 |
| Source/Internet/Access | Accessibility of the internet source | 2004 |
| Source/Internet/Attitude/Trust | Trust incorporates reliability, credibility, perceived quality, and confidence towards an internet source of information. | 2007 |
| Source/Internet/Beliefs | The cognitive act or state in which a proposition about the internet is taken to be true | 2008 |
| Source/Internet/Experience | Previous exposure to the internet source of information | 2003 |
| Source/Internet/Type | Type of internet information source | 2010 |
| Source/Knowledge | The level of knowledge an individual possesses about an information source. | 2013 |
| Source/Non-healthcare/Attitude/Trust | Trust incorporates reliability, credibility, perceived quality, and confidence towards a non-healthcare information source. | 2007 |
| Source/Non-healthcare/Experience | Previous exposure to a non-healthcare information source | 2009 |
| Source/Non-healthcare/Type | Type of non-healthcare information source | 1999 |
| Unclassified | Variable not listed elsewhere or in any other category. | 2010 |

### Table 5: Network Statistics

Table S5: Network statistics for the complete model for all years, up to 2008 (inclusive) and post-2008.

| Network Statistic | All Terms | Pre and including 2008 | Post 2008 | Pre and including 2014 | Post 2014 |
| --- | --- | --- | --- | --- | --- |
| Nodes | 67 | 52 | 66 | 67 | 66 |
| Edges | 4128 | 468 | 3660 | 2115 | 2013 |
| Node properties | | | | | |
| Mean Degree | 123.224 | 18.000 | 110.909 | 63.134 | 61.000 |
| Mean Closeness | 0.665 | 0.521 | 0.659 | 0.373 | 0.593 |
| Mean Betweenness | 17.806 | 24.808 | 18.030 | 21.716 | 23.667 |
| Eigenvector centrality | 0.154 | 0.213 | 0.154 | 0.152 | 0.157 |
| Network properties | | | | | |
| Network Diameter | 3 | 4 | 3 | 4 | 4 |
| Mean Distance | 11.540 | 1.973 | 1.555 | 1.678 | 1.728 |
| Edge Density/Graph Density | 1.867 | 0.353 | 1.706 | 0.957 | 0.938 |
| Reciprocity | 1 | 1 | 1 | 1 | 1 |
| Transitivity | 0.652 | 0.515 | 0.641 | 0.590 | 0.558 |
| Connected Components | 1 | 1 | 1 | 2 | 1 |
| Modularity | 3 | 6 | 2 | 5 | 3 |

### Network Analysis Methods

Node location in relation to each other node was determined by force-directed placement of the Fruchterman-Reingold layout algorithm. This algorithm did not take into consideration the weights of the edges and positions the nodes on a map based on the connection strength. That is, nodes with higher weights (co-occurrence weights) and/or more connections to other nodes are located more commonly in the center of the network map. Whereas, nodes with lower weights are connected on the periphery.^1,2^ The layout was chosen as it provided a clean area spread. The Force Atlas algorithms are more commonly used but did not provide networks that could be used to compare to each other, like the spherical structure of Fruchtermann-Reingold.^3,4^

References

1. Fruchterman TM, Reingold EM. Graph drawing by force‐directed placement. *Software: Practice and experience.* 1991;21(11):1129-1164.

2. Mullarkey MC, Marchetti I, Beevers CG, et al. Using Network Analysis to Identify Central Symptoms of Adolescent Depression Using Network Analysis to Identify Central Symptoms of Adolescent Depression. 2019.

3. Cherven K. *Mastering Gephi network visualization.* Packt Publishing Ltd; 2015.

4. Khokhar D. *Gephi cookbook.* Packt Publishing Ltd; 2015.
